# Supplementary material for: Statin treatment effectiveness and the SLCO1B1*5 reduced function genotype: Long‐term outcomes in women and men
Source: Br J Clin Pharmacol. 2022 Feb 14;88(7):3230–40. doi: 10.1111/bcp.15245 (PMC9305522; doi:10.1111/bcp.15245)
Supplement: Supplementary file 1 — FIGURE S1 Uncensored Kaplan‐Meier plots for effect of rs4149056 C genotype (SLCO1B1*5) on discontinuation of atorvastatin and simvastatin in UK Biobank primary care data TABLE S1 Expanded descriptive summary statistics table for UK Biobank participants included in analysis TABLE S2 Expanded SLCO1B1 genotype associations with baseline analyses in patients who reported statin treatment TABLE S3 SLCO1B1*5 genotype association with discontinuing simvastatin and atorvastatin treatment in the GP prescribing data TABLE S4 Switching to another statin from atorvastatin or simvastatin, within 12 months of discontinuation TABLE S5 Last recorded dose of GP‐prescribed simvastatin or atorvastatin in the participants who discontinued treatment TABLE S6 GP‐diagnosed muscle symptoms by SLCO1B1*5 genotype and sex, stratified by stable treatment period (3 months after first prescription) [file BCP-88-3230-s001.docx]

**Statin treatment effectiveness and the SLCO1B1*5 reduced function genotype: long-term outcomes in women and men**

Türkmen *et al.* 2022

**Supplementary Information**

[Supplementary Figure 1 2](#_Toc78546487)

[Supplementary Table 1 3](#_Toc78546488)

[Supplementary Table 2 4](#_Toc78546489)

[Supplementary Table 3 6](#_Toc78546490)

[Supplementary Table 4 7](#_Toc78546491)

[Supplementary Table 5 8](#_Toc78546492)

[Supplementary Table 6 9](#_Toc78546492)

# Supplementary Figure 1

Uncensored Kaplan-Meier plots for effect of rs4149056 C genotype (*SLCO1B1* *5) on discontinuation of atorvastatin and simvastatin in UK Biobank primary care data


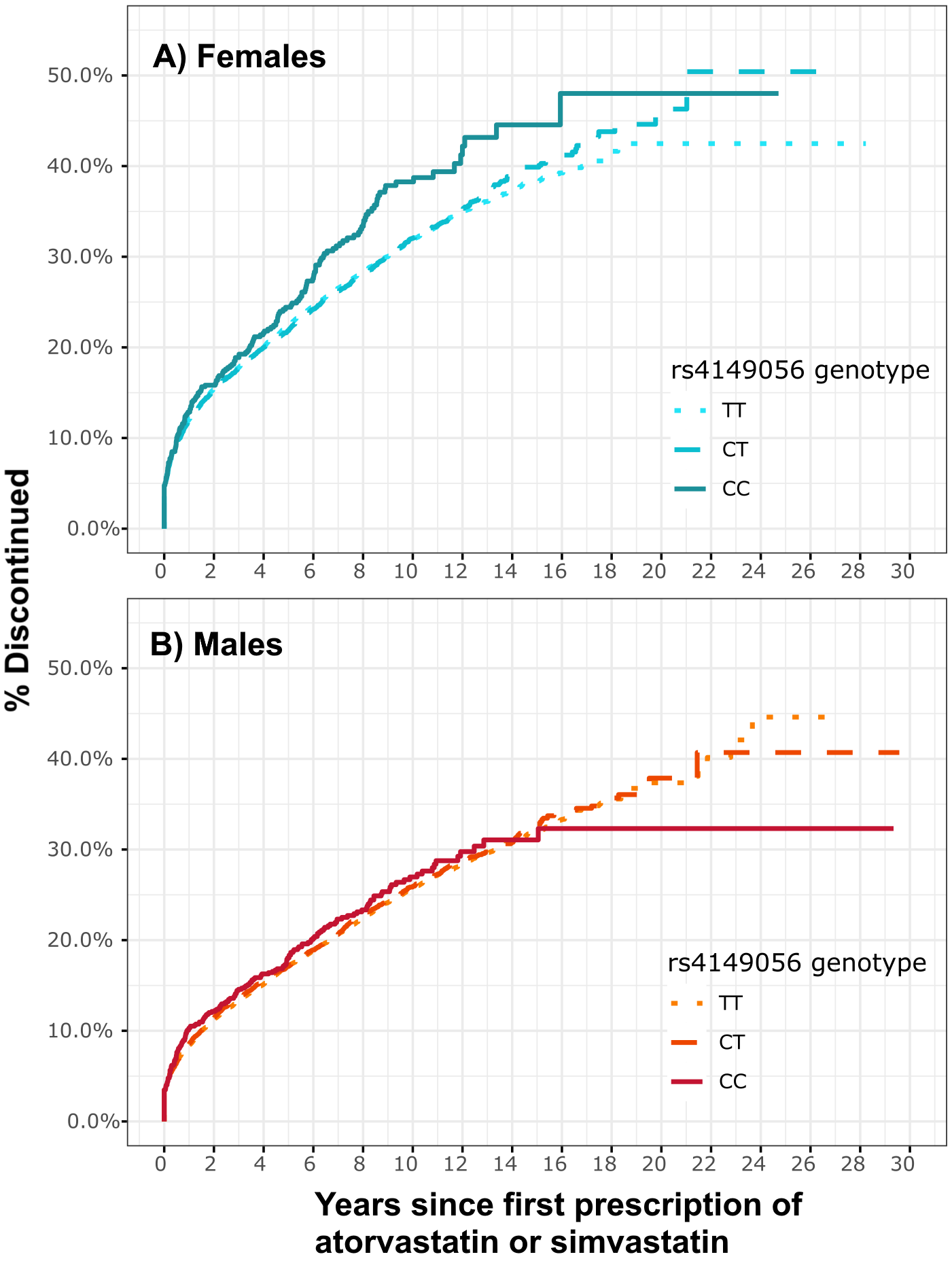


Genetic variant rs4149056 (*SLCO1B1* *5) C-allele associations with discontinuing GP-prescribed simvastatin or atorvastatin treatment in males and females separately. Plots show the cumulative incidence over time of discontinuing treatment in females (A) and males (B), stratified by rs4149056 genotype.

# Supplementary Table 1

Expanded descriptive summary statistics table for UK Biobank participants included in analysis

|  |  | **rs4149056 (*SLCO1B1* *5) genotype** | | | | | |
| --- | --- | --- | --- | --- | --- | --- | --- |
|  |  | **Female** | | | **Male** | | |
|  |  | **TT (*1/*1) homozgotes** | **TC (*1/*5) heterozygotes** | **CC (*5/*5) homozygotes** | **TT (*1/*1) homozgotes** | **TC (*1/*5) heterozygotes** | **CC (*5/*5) homozygotes** |
| ***- Baseline assessment (self-reported)*** | |  |  |  |  |  |  |
| n (% of genotype group) |  | 18,925(72.27) | 6,669(25.47) | 591(2.26) | 29,996(72.38) | 10,522(25.39) | 927(2.24) |
| Age | Min-max | 40-70 | 40-70 | 41-70 | 40-70 | 40-70 | 40-70 |
|  | Mean (SD) | 61.7(5.7) | 61.5(5.7) | 61.6(5.8) | 61.4(6.1) | 61.3(6) | 61.6(6.1) |
| Weight | Mean (SD) | 76.3 (15.4) | 76.2 (15.2) | 76.3 (15.7) | 89.3(15.2) | 89.2(15.0) | 89.8(14.9) |
| BMI | Mean (SD) | 29.5(5.6) | 29.4(5.6) | 29.5(5.6) | 29.31(4.5) | 29.3(4.4) | 29.4(4.3) |
| LDL, n >3mmol/L (% of genotype group) | | 6,492(36.17) | 2,367(37.18) | 250(44.8) | 7,743(27.1) | 2,845(28.46) | 267(30.2)) |
| Triglycerides, n >2.3mmol/L (%) | | 5,040(26.63) | 1,831(27.46) | 178(30.12) | 9,841(32.81) | 3,660(34.78) | 345(37.22) |
| Total cholesterol, n >5mmol/L (%) | | 7,485(41.65) | 2,730(42.80) | 270(48.39) | 7,069(24.7) | 2,668(26.64) | 258(29.05) |
| HbA1c, n >47mmol/mol (%) | | 2,526(13.99) | 889(14) | 105(18.52) | 4,558(15.94) | 1,572(15.74) | 124(13.98) |
| ***- Primary Care data*** |  |  |  |  |  | | |
| n (% of genotype group) |  | 21,345(72.17) | 7,538(25.49) | 691(2.34) | 28,608(72.22) | 10,056(25.39) | 947(2.39) |
| Age at first statin prescription | Min-max | 40-78.9 | 40-79.1 | 40.3-77.31 | 40-79.2 | 40-79.1 | 41.1-78.2 |
|  | Mean (SD) | 61,9 (7.1) | 61.8(7.1) | 61.9(7.3) | 60.9 (7.2) | 60.8(7.2) | 61.1(6.9) |
| Years between first and last statin* | Min-max | 0.002-28.2 | 0.002-26.3 | 0.01-24.7 | 0.002-27.2 | 0.002-29.5 | 0.01-29.3 |
|  | Mean (SD) | 5.7(4.8) | 5.7(4.7) | 5.4(4.5) | 6.6 (4.8) | 6.6(4.9) | 6.6(4.9) |
| Muscle diagnoses prior to statin* | n (%) | 560(2.62) | 190(2.52) | 19(2.75) | 499(1.74) | 154(1.53) | 19(2.01) |
| MI/angina diagnoses prior to statin* | n (%) | 1,078(5.05) | 400(5.31) | 36(5.21) | 3,151(11.01) | 1,158(11.52) | 107(11.3) |
| Muscle diagnoses after first statin* | n (%) | 776(3.64) | 312(4.14) | 26(3.76) | 880(3.08) | 314(3.12) | 34(3.59) |
| MI/angina after first statin* | n (%) | 2,875(13.47) | 995(13.20) | 98(14.18) | 6,978(24.39) | 2,431(24.17) | 211(22.28) |
| Discontinuation ever, n (%) | n (%) | 5,476(25.65) | 1,946(25.82) | 204(29.52) | 6,119(21.39) | 2,182(21.7) | 212(22.39) |
| Discontinuation in 1 year, n (%) | n (%) | 2,489(11.66) | 865(11.48) | 86(12.45) | 2,333(8.15) | 842(8.37) | 95(10.03) |
| Discontinuation in year 1+, n (%) | n (%) | 2,987(17.62) | 1,081(18.09) | 118(21.77) | 3,786(15.49) | 1,340(15.59) | 117(14.53) |

* simvastatin or atorvastatin prescription

# Supplementary Table 2

Expanded SLCO1B1 genotype associations with baseline analyses in patients who reported statin treatment

|  |  |  | **Female** | |  |  |  | **Male** |  |  |  |
| --- | --- | --- | --- | --- | --- | --- | --- | --- | --- | --- | --- |
| **Outcomes** | **SLCO1B1 genotype** | **N cases (%of genotype)** | **Odds ratio** | **95% CIs** | | **p-value** | **N cases** | **Odds ratio** | **95% CIs** | | **p-value** |
| **Total cholesterol** | TT (*1/*1) | 7,485(41.65) | REF |  |  |  | 7,069(24.7) | REF |  |  |  |
|  | TC (*1/*5) | 2,730(42.80) | 1 | 0.99 | 1.1 | 0.131 | 2,668(26.64) | 1.11 | 1.05 | 1.16 | <0.005 |
|  | CC (*5/*5) | 270(48.39) | 1.3 | 1.1 | 1.6 | 0.001 | 258(29.05) | 1.27 | 1.09 | 1.47 | 0.001 |
| **LDL** | TT (*1/*1) | 6,492(36.17) | REF |  |  |  | 7,743(27.1) | REF |  |  |  |
|  | TC (*1/*5) | 2,367(37.18) | 1 | 0.98 | 1.1 | 0.185 | 2,845(28.46) | 1.07 | 1.01 | 1.12 | 0.01 |
|  | CC (*5/*5) | 250(44.8) | 1.4 | 1.2 | 1.7 | 4.4 x 10-5 | 267(30.2) | 1.18 | 1.02 | 1.37 | 0.025 |
| **Triglycerides** | TT (*1/*1) | 5,040(26.63) | REF |  |  |  | 9,841(32.81) | REF |  |  |  |
|  | TC (*1/*5) | 1,831(27.46) | 1.1 | 1.01 | 1.2 | 0.024 | 3,660(34.78) | 1.09 | 1.04 | 1.15 | 0.004 |
|  | CC (*5/*5) | 178(30.12) | 1.2 | 0.98 | 1.4 | 0.078 | 345(37.22) | 1.26 | 1.1 | 1.46 | 0.001 |
| **Headache** | TT (*1/*1) | 1,807 (9.58) | REF |  |  |  | 1,523 (5.09) | REF |  |  |  |
|  | TC (*1/*5) | 608 (9.14) | 0.9 | 0.85 | 1 | 0.233 | 512 (4.87) | 0.95 | 0.86 | 1.06 | 0.39 |
|  | CC (*5/*5) | 75 (12.71) | 1.4 | 1.08 | 1.8 | 0.01 | 56 (6.04) | 1.22 | 0.92 | 1.61 | 0.16 |
| **Fatigue/tiredness** | TT (*1/*1) | 3,017 (16.56) | REF |  |  |  | 3,676 (12.64) | REF |  |  |  |
|  | TC (*1/*5) | 1,088 (16.97) | 1 | 0.94 | 1.1 | 0.739 | 1,386 (13.59) | 1.08 | 1.01 | 1.16 | 0.018 |
|  | CC (*5/*5) | 93 (16.26) | 1 | 0.76 | 1.2 | 0.737 | 111 (12.35) | 0.98 | 0.8 | 1.2 | 0.872 |
| **Pain** | TT (*1/*1) | 9,741(51.47) | REF |  |  |  | 13,184(43.95) | REF |  |  |  |
|  | TC (*1/*5) | 3,448(51.7) | 1 | 0.95 | 1.1 | 0.811 | 4,660(44.29) | 1.01 | 0.97 | 1.06 | 0.575 |
|  | CC (*5/*5) | 289(48.9) | 0.9 | 0.76 | 1.1 | 0.203 | 409(44.12) | 1 | 0.88 | 1.15 | 0.918 |
| **Hba1c** | TT (*1/*1) | 2,526(13.99) | REF |  |  |  | 4,558(15.94) | REF |  |  |  |
|  | TC (*1/*5) | 889(14) | 1 | 0.92 | 1.1 | 0.933 | 1,572(15.74) | 0.98 | 0.92 | 1.05 | 0.6 |
|  | CC (*5/*5) | 105(18.52) | 1.4 | 1.13 | 1.8 | 0.002 | 124(13.98) | 0.86 | 0.71 | 1.04 | 0.12 |
| **CRP level** | TT (*1/*1) | 1,937 (10.24) | REF |  |  |  | 2,542 (8.47) | REF |  |  |  |
|  | TC (*1/*5) | 665 (9.97) | 1.1 | 0.95 | 1.2 | 0.234 | 986 (9.37) | 1.15 | 1 | 1.25 | 0.058 |
|  | CC (*5/*5) | 80 (13.54) | 1.5 | 1.05 | 2 | 0.022 | 76 (8.20) | 0.94 | 0.66 | 1.35 | 0.757 |
| **ALT level** | TT (*1/*1) | 5,867 (32.64) | REF |  |  |  | 7,747 (27.07) | REF |  |  |  |
|  | TC (*1/*5) | 2,101 (32.93) | 1 | 0.95 | 1.1 | 0.753 | 2,720 (27.18) | 1 | 0.95 | 1.05 | 0.993 |
|  | CC (*5/*5) | 207 (37.10) | 1.2 | 1.01 | 1.4 | 0.033 | 249 (28.10) | 1.07 | 0.92 | 1.25 | 0.358 |

# Supplementary Table 3

*SLCO1B1* *5 genotype association with discontinuing simvastatin and atorvastatin treatment in the GP prescribing data

|  | **Sex** | ***SLCO1B1* genotype** | **N** | **N disc*** | **Person-years** | **Discontinuations per 100 statin-years** | **HR** | **95% CIs** | | ***p*** |
| --- | --- | --- | --- | --- | --- | --- | --- | --- | --- | --- |
| Discontinued | Female | TT (*1/*1) | 21,347 | 2,489 | 18,290 | 13.6 | *ref* |  |  |  |
| atorvastatin and |  | TC (*1/*5) | 7,538 | 865 | 6,466 | 13.4 | 0.98 | 0.91 | 1.06 | 0.63 |
| simvastatin |  | CC (*5/*5) | 691 | 86 | 589 | 14.6 | 1.06 | 0.86 | 1.32 | 0.55 |
| treatment |  | *Total* | *29,576* | *3,440* | *25,345* | *13.6* |  |  |  |  |
| <1 year after |  |  |  |  |  |  |  |  |  |  |
| first prescription | Male | TT (*1/*1) | 28,609 | 2,333 | 25,743 | 9.1 | *ref* |  |  |  |
|  |  | TC (*1/*5) | 10,056 | 842 | 9,054 | 9.3 | 1.03 | 0.95 | 1.1 | 0.48 |
|  |  | CC (*5/*5) | 947 | 95 | 846 | 11.2 | 1.25 | 1.02 | 1.53 | 0.03 |
|  |  | *Total* | *39,612* | *3,270* | *35,644* | *9.2* |  |  |  |  |
| Discontinued | Female | TT (*1/*1) | 16,950 | 2,987 | 105,435 | 2.8 | *ref* |  |  |  |
| atorvastatin and |  | TC (*1/*5) | 5,977 | 1,081 | 37,192 | 2.9 | 1.03 | 0.96 | 1.1 | 0.41 |
| simvastatin |  | CC (*5/*5) | 542 | 118 | 3,198 | 3.7 | 1.3 | 1.08 | 1.56 | 0.01 |
| treatment |  | *Total* | *23,469* | *4,186* | *145,824* | *2.9* |  |  |  |  |
| >1 years after |  |  |  |  |  |  |  |  |  |  |
| first prescription | Male | TT (*1/*1) | 24,440 | 3,786 | 163,312 | 2.3 | *ref* |  |  |  |
|  |  | TC (*1/*5) | 8,594 | 1,340 | 57,588 | 2.3 | 1 | 0.94 | 1.07 | 0.88 |
|  |  | CC (*5/*5) | 805 | 117 | 5,442 | 2.1 | 0.94 | 0.78 | 1.12 | 0.48 |
|  |  | *Total* | *33,839* | *5,243* | *226,342* | *2.3* |  |  |  |  |
| Discontinued | Female | TT (*1/*1) | 21,347 | 5,476 | 123,725 | 4.4 | *ref* |  |  |  |
| atorvastatin and |  | TC (*1/*5) | 7,538 | 1,946 | 43,657 | 4.5 | 1 | 0.96 | 1.06 | 0.79 |
| simvastatin |  | CC (*5/*5) | 691 | 204 | 3,787 | 5.4 | 1.19 | 1.03 | 1.37 | 0.01 |
| treatment |  | *Total* | *29,576* | *7,626* | *171,169* | *4.5* |  |  |  |  |
| (any time) |  |  |  |  |  |  |  |  |  |  |
|  | Male | TT (*1/*1) | 28,609 | 6,119 | 189,055 | 3.2 | *ref* |  |  |  |
|  |  | TC (*1/*5) | 10,056 | 2,182 | 66,642 | 3.3 | 1.01 | 0.96 | 1.06 | 0.58 |
|  |  | CC (*5/*5) | 947 | 212 | 6,289 | 3.4 | 1.05 | 0.92 | 1.2 | 0.44 |
|  |  | *Total* | *39,612* | *8,513* | *261,986* | *3.2* |  |  |  |  |

Discontinuations = discontinued both simvastatin and atorvastatin prior to data censoring date

# Supplementary Table 4

Switching to another statin from atorvastatin or simvastatin, within 12 months of discontinuation

|  | ***rs4149056 (SLCO1B1 *5) genotype*** | | | | | |
| --- | --- | --- | --- | --- | --- | --- |
|  | **Female** | | | **Male** | | |
|  | **TT (*1/*1)** | **TC (*1/*5)** | **CC (*5/*5)** | **TT (*1/*1)** | **TC (*1/*5)** | **CC (*5/*5)** |
| Pravastatin | 886 | 316 | 26 | 962 | 332 | 34 |
| Fluvastatin | 40 | 13 | 3 | 39 | 16 | 4 |
| Rosuvastatin | 628 | 242 | 25 | 707 | 263 | 21 |
| Cerivastatin | 1 | 0 | 0 | 3 | 0 | 0 |
| *Total* | *1328* | *502* | *50* | *1525* | *540* | *55* |
|  |  |  |  |  |  |  |
| Discontinued | 5,476 | 1,946 | 204 | 6,119 | 2,182 | 212 |
|  |  |  |  |  |  |  |
| % switched | 24.3 | 25.8 | 24.5 | 24.9 | 24.7 | 25.9 |

Total = number of participants in each sex/genotype group who received a prescription to any another statin within 12 months of their last atorvastatin/simvastatin (some received more than one statin, so the total number of individual statins is greater than the number of people).

# Supplementary Table 5

Last recorded dose of GP-prescribed simvastatin or atorvastatin in the participants who discontinued treatment

|  | ***rs4149056 (SLCO1B1 *5) genotype*** | | | | | |
| --- | --- | --- | --- | --- | --- | --- |
|  | **Female** | | | **Male** | | |
|  | **TT (*1/*1)** | **TC (*1/*5)** | **CC (*5/*5)** | **TT (*1/*1)** | **TC (*1/*5)** | **CC (*5/*5)** |
| Simvastatin |  |  |  |  |  |  |
| 10 mg | 405(10.92) | 136(10.99) | 14(9.21) | 360(8.06) | 109(7.38) | 9(7.09) |
| 20 mg | 1256(33.85) | 424(34.28) | 51(33.55) | 1224(27.39) | 398(26.96) | 39(30.71) |
| 40 mg | 2031(54.74) | 677(54.73) | 85(55.92) | 2858(63.97) | 956(64.77) | 79(62.20) |
| 80 mg | 18(0.49) | 0 | 2(1.32) | 26(0.58) | 13(0.88) | 0 |
| *Total* | *3,710* | *1,237* | *152* | *4,468* | *1,476* | *127* |
| Atorvastatin |  |  |  |  |  |  |
| 10 mg | 1,161(48.93) | 423(50.3) | 37(52.86) | 1075(41.73) | 388(45.33) | 32(33.68) |
| 20 mg | 805 (33.92) | 281(33.41) | 27(38.57) | 929(36.06) | 290(33.88) | 37(38.95) |
| 40 mg | 336 (14.16) | 113(13.4) | 6(8.57) | 454(17.62) | 138(16.12) | 23(24.21) |
| 80 mg | 71(2.99) | 24(2.85) | 0 | 118(4.58) | 40(4.67) | 3(3.16) |
| *Total* | *2,373* | *841* | *70* | *2,576* | *856* | *95* |

# Supplementary Table 6

GP-diagnosed muscle symptoms by SLCO1B1 *5 genotype and sex, stratified by stable treatment period (3 months after first prescription)

| **Model** | **Sex** | **Genotype** | **N cases (%)** | **HR** | **95% CIs** | | ***p*** |
| --- | --- | --- | --- | --- | --- | --- | --- |
| Within | Female |  |  |  |  |  |  |
| 3 months |  | TT (*1/*1) | 74 (0.36) | *ref* |  |  |  |
| of first |  | CT (*1/*5) | 34(0.48) | 1.31 | 0.9 | 2 | 0.18 |
| prescription |  | CC (*5/*5) | 2(0.3) | 0.86 | 0.2 | 3.5 | 0.82 |
|  |  | *Total* | *110* |  |  |  |  |
|  | Male |  |  |  |  |  |  |
|  |  | TT (*1/*1) | 69(0.25) | *ref* |  |  |  |
|  |  | CT (*1/*5) | 25(0.26) | 1.03 | 0.7 | 1.6 | 0.9 |
|  |  | CC (*5/*5) | 2(0.22) | 0.88 | 0.2 | 3.7 | 0.86 |
|  |  | *Total* | *96* |  |  |  |  |
| Greater than | Female |  |  |  |  |  |  |
| 3 months |  | TT (*1/*1) | 584(3.10) | *ref* |  |  |  |
| after first |  | CT (*1/*5) | 244(3.67) | 1.19 | 1 | 1.4 | 0.02 |
| prescription |  | CC (*5/*5) | 20(3.31) | 1.11 | 0.7 | 1.7 | 0.64 |
|  |  | *Total* | *848* |  |  |  |  |
|  | Male |  |  |  |  |  |  |
|  |  | TT (*1/*1) | 736(2.81) | *ref* |  |  |  |
|  |  | CT (*1/*5) | 262(2.85) | 1.01 | 0.9 | 1.2 | 0.85 |
|  |  | CC (*5/*5) | 28(3.22) | 1.16 | 0.8 | 1.7 | 0.43 |
|  |  | *Total* | *1,026* |  |  |  |  |
